# Rationale for the Use of Acupuncture to Stabilize Blood Pressure Fluctuations During Total Laparoscopic Hysterectomy: Protocol for a Pilot Parallel-Group Randomized Clinical Trial

**DOI:** 10.2196/77009

**Published:** 2025-07-22

**Authors:** Jee Young Lee, Ju-Won Roh, Kyung-Hee Han, Min-Jeong Kim, Young Jeong Na, Bo Seong Yun, Joohyun Lee

**Affiliations:** 1 Department of Korean Medicine, Integrative Cancer Center CHA Ilsan Medical Center CHA University Goyang-si Republic of Korea; 2 Department of Obstetrics and Gynecology CHA Ilsan Medical Center CHA University Goyang-si Republic of Korea; 3 Seoul National University Hospital Seoul Republic of Korea; 4 Department of Anesthesia and Pain Medicine CHA Ilsan Medical Center CHA University Goyang-si Republic of Korea

**Keywords:** general anesthesia, blood pressure, hemodynamic, acupuncture, hysterectomy, laparoscopy, leiomyoma

## Abstract

**Background:**

Reducing blood pressure fluctuations during surgery is a key objective in improving patient outcomes. Although acupuncture has been suggested as a potential noninvasive intervention for blood pressure modulation, its effectiveness in reducing intraoperative fluctuations remains unclear.

**Objective:**

This study aims to investigate whether acupuncture can help stabilize blood pressure during surgery, particularly in women undergoing laparoscopic hysterectomy, a procedure known to cause marked hemodynamic changes during the early intraoperative phase.

**Methods:**

This is a prospective, single-center, randomized controlled clinical trial with a parallel-group design. Forty-eight adult patients scheduled to undergo a total laparoscopic hysterectomy are eligible for this study. Participants who provide consent will be randomly assigned in a 1:1 ratio to the acupuncture or placebo groups. They will be followed up for at least 14 days to assess the safety of the intervention, general anesthesia, and surgery. Researchers will compare the difference between the highest and lowest mean blood pressures from anesthesia induction to the postincision period as the primary endpoint. As secondary outcomes, systolic, diastolic, and mean blood pressures will be compared at each predetermined timepoint. The incidences of hypotension, hypertension, tachycardia, and bradycardia will be determined separately. The use of remifentanil at the early stage of surgery, rate of surgical discontinuation, and length of hospital stay will be assessed as surrogate indicators of stable general anesthesia and surgical procedures. For patient-reported outcomes, the Spielberger State-Trait Anxiety Inventory and EQ-5D-5L will be used to evaluate changes in anxiety and overall quality of life. This study may support the use of acupuncture as a complementary intervention to help maintain hemodynamic stability during laparoscopic hysterectomy. The study will be conducted in accordance with the Declaration of Helsinki and has been approved by the Institutional Review Board of CHA Ilsan Medical Center (ICHA 2022-11-010; date of approval: January 03, 2023).

**Results:**

Recruitment began in October 2023 and is expected to continue until December 2025. The intervention and data collection procedures are proceeding without major difficulties, with a dropout rate of 11.4%. As of June 2025, a total of 35 participants have been successfully recruited and randomized. Final data analysis is scheduled for completion by March 2026, and the study results are expected to be published in December 2026.

**Conclusions:**

This trial will provide valuable evidence on the efficacy of acupuncture for stabilizing intraoperative blood pressure and supporting hemodynamic control during laparoscopic hysterectomy.

**Trial Registration:**

ClinicalTrials.gov NCT05720884; https://clinicaltrials.gov/study/NCT05720884

**International Registered Report Identifier (IRRID):**

DERR1-10.2196/77009

## Introduction

Various factors, such as the use of agents that induce anesthesia, surgery-induced stress, and hemorrhage, contribute to blood pressure fluctuations during surgery. Because blood pressure values lower or higher than the normal range can affect a patient’s prognosis, reducing blood pressure fluctuations during surgery is an important goal for anesthesiologists [1,2]. A previous study reported that both hypotension and hypertension increased the oxygen demand in peripheral organs, postoperative complications, and mortality [3].

Blood pressure fluctuations that occur during anesthesia induction, tracheal intubation, and the early stages of surgery can develop rapidly within a short time due to the dynamic balance between anesthetic administration and noxious surgical stimuli. Hypotension may result from excessive analgesic or anesthetic dosing, while hypertension may arise from insufficient depth of anesthesia or sudden intense stimulation. Therefore, it is essential to anticipate the timing and intensity of such stimuli and preemptively titrate the concentration of analgesic agents to maintain hemodynamic stability. To minimize these abrupt hemodynamic changes, anesthesiologists and surgeons must perform thorough preoperative evaluations and develop individualized anesthetic plans tailored to the surgical context and patient condition [4-6].

Acupuncture is an established adjunctive therapy in anesthetic practice. Numerous studies have demonstrated that it can reduce the required doses of anesthetic and opioid agents, and alleviate postoperative symptoms such as pain, ileus, nausea, and vomiting [7]. These effects are thought to be mediated through modulation of the autonomic nervous system and nociceptive pathways, ultimately contributing to greater physiological stability throughout the perioperative period. However, despite these established effects, the role of acupuncture in directly modulating intraoperative blood pressure remains unexplored. The few available studies have primarily addressed either essential hypertension or the prevention of anesthesia-related hypotension as isolated conditions [8,9].

Notably, acupuncture has demonstrated bidirectional regulatory potential, with the capability of lowering elevated blood pressure and elevating suppressed blood pressure depending on the physiological context [10-13]. This dual effect suggests that acupuncture may serve as a stabilizing intervention for both extremes of hemodynamic instability under general anesthesia.

To investigate this, the study group designed a randomized controlled clinical trial to assess the impact of acupuncture on blood pressure fluctuations in patients undergoing total laparoscopic hysterectomy, a gynecological procedure known to cause significant hemodynamic shifts during the early intraoperative phase [14,15]. The study group hypothesized that acupuncture may contribute to overall blood pressure stability by attenuating both hypertensive and hypotensive responses in this highly dynamic setting.

## Methods

### Study Design

This is a prospective, single-center, randomized controlled clinical trial with a parallel-group design for blood pressure fluctuations during general anesthesia. Forty-eight adult patients scheduled to undergo a total laparoscopic hysterectomy will be included in this study. Participants who voluntarily consent to participate in the clinical trial will be randomly assigned in a 1:1 ratio to the acupuncture or placebo group. Eligible participants will be followed up for at least 14 days to assess the safety of the intervention, general anesthesia, and surgery.

The study protocol adheres to both the SPIRIT (Standard Protocol Items: Recommendations for Interventional Trials) guidelines (Multimedia Appendix 1) and STRICTA (Standards for Reporting Interventions in Clinical Trials of Acupuncture) guidelines (Multimedia Appendix 2). All study procedures will be performed in accordance with the Declaration of Helsinki and Korean Good Clinical Practice Guidelines. The study protocol has been registered and updated at ClinicalTrials.gov (NCT05720884), the Clinical Research Information Service, and the South Korean registration service for clinical trials (KCT0009149). This article describes the study based on protocol version 1.4 (dated November 13, 2024). The latest protocol will be updated in a timely manner for both registration sites.

### Ethical Considerations

The study protocol has been approved by the Institutional Review Board of CHA Ilsan Medical Center (ICHA 2022-11-010; date of approval: January 03, 2023). Written informed consent will be obtained from all participants after a thorough explanation of the study’s purpose and procedures, and after confirming their voluntary willingness to participate. To protect personal information, all data will be anonymized and stored using initials. Participants will receive a total compensation of 60,000 KRW (approximately US $45) for their time and involvement. Their participation may contribute to reducing perioperative blood pressure fluctuations for the broader public benefit.

This clinical study involves a data and safety monitoring board (DSMB) consisting of at least one medical clinician and a statistician. The composition and operation of the DSMB will be in accordance with the standard operating procedures of the trial. Study monitoring is scheduled to be conducted once a year. If a protocol modification is recommended, an appropriate amendment or response will be provided. If a suspected unexpected serious adverse reaction case occurs or the DSMB suspects a significant harmful association with the intervention and requests unblinding, the intervention researcher will unblind the allocation to other researchers and the committee, and submit the unblinded interim results.

### Eligibility

Participants scheduled to undergo surgical removal of the uterus for uterine fibroids and those who have an expected date of total laparoscopic hysterectomy by a gynecologist are eligible. The specific inclusion criteria are as follows: (1) age between 19 and 69 years, (2) American Society of Anesthesiologists (ASA) class I or II, (3) requirement for surgical removal of the uterus by a gynecology specialist, and (4) scheduled to undergo total laparoscopic hysterectomy in advance. All participants will be required to understand and agree with the study protocol. A delegated researcher or research coordinator will obtain written informed consent from all participants.

Participants under the following conditions or situations are ineligible: (1) survival expectancy of less than 3 months; (2) emergency operation, which was not scheduled in advance; (3) hypertension or hypotension that can significantly interfere with the study result; (4) diagnosis of arrhythmia, such as atrial fibrillation and frequent ventricular or supraventricular premature beats; (5) diagnosis of heart failure or valvular disease; (6) anemia with hemoglobin level <12 g/dL; (7) incapable of surgery due to hemodynamic or medical reasons other than stated above; (8) incapable of receiving acupuncture treatment on the determined location; (9) current use of gonadotropin-releasing hormone receptor agonists; (10) current use of drugs that may interfere with the result, including steroids, immunosuppressants, and drugs for psychiatric disorders; (11) significant comorbidities that may interfere with the interpretation of intervention efficacy or results, such as stroke, myocardial infarction, kidney disease, dementia, or epilepsy; (12) pregnant, planning to be pregnant within the study period, or breastfeeding; (13) previous participation in any other clinical trial within 1 month of participation, planning to participate in another clinical trial within 6 months after the enrollment date, or planning to participate in another clinical trial in the follow-up period; (14) failure to complete the informed consent form voluntarily; and (15) being deemed to be unsuitable for participation.

### Randomization and Allocation

Eligible participants who sign the informed consent form will be randomly assigned to the acupuncture or placebo group in a 1:1 ratio. Random sequences will be generated by permuted block randomization of block size 2 or 4 using the random allocation tool in R software 4.1 (R Foundation for Statistical Computing). Random sequence generation will be performed, and random sequences will be determined by an independent statistician prior to enrollment. Only the statistician who generates the random sequence will be aware of the full randomization sequence.

Each random sequence will be sealed in an opaque envelope and stored in a double-locked cabinet. The delegated researcher or research coordinator will explain the trial process to the candidate, obtain written informed consent, open the randomization envelope for each participant, and assign them to an appropriate group.

Intervention researchers will perform acupuncture or placebo acupuncture according to their assigned group. The intervention researchers will stay unblinded because the researchers will perform different interventions according to the group. However, the researchers will separate the person and location to maintain anonymization as robustly as possible. The participant, assessor, and statistician will be blinded, and the location where the intervention is performed will be separate from the location where the assessment will be performed. Interventions will be performed in the ward, and assessments will be performed in the operating room.

### Intervention

Acupuncture will be performed with a disposable, sterilized, stainless steel needle measuring 0.20 × 30 mm (Dongbang Medical) in both groups. Ten acupoints (PC6, PC5, SP3, KI3, and ST36) will be chosen bilaterally for the intervention group (acupuncture group). The depth of acupuncture varies (1 to 1.5 cm) among the participants. After acupuncture needles are inserted, they will be manually manipulated to obtain a predetermined sensation, De Qi. Acupoints PC6 and PC5 will be inserted with depth adjustment to reach the anatomical plane right above the median nerve, thereby facilitating a more prominent sensation. Acupuncture will continue for 20 minutes. Ten other points that are not official acupoints and are not on the median or peroneal nerve will be chosen for the placebo group. An acupuncture needle depth of 0.5 cm will be used.

Acupuncture or placebo will be administered 2 times during the first and second visits. Administration will be done by a qualified acupuncturist who is a physician in Korean medicine with 10 years of clinical experience, independent of the anesthesia team.

### Study Process

After a sufficient explanation of the purpose and methods of this clinical study by a researcher, subjects who voluntarily decide to participate and sign a written consent form will be evaluated for eligibility at the screening visit. Eligible subjects will be randomly assigned to the acupuncture or placebo control group according to randomization. Acupuncture treatment will be performed twice, one day before surgery (V1) and on the day of surgery (V2).

All intraoperative management and outcome assessments will be independently performed by anesthesiologists who are not involved in the acupuncture procedure. All participants will fast for 6-8 hours prior to surgery. After entering the operating room (V3), electrocardiography, pulse oximetry, and monitoring of the partial pressure of end-tidal carbon dioxide (IntelliVue MX550; Phillips North America Corporation) will be performed continuously. Automated oscillometric measurements of blood pressure at 2.5-minute intervals and “train of four” (TOF) (ToFscan; Drager Technologies) assessments will also be performed. Throughout the surgery, these data will be downloaded to personal computers using RS232C cables.

After checking the initial vital signs (heart rate, blood pressure, and oxygen saturation) (P1), anesthesia will be induced intravenously with propofol (1-2.5 mg/kg) and remifentanyl (0.01-0.1 ng/kg/min). Mask ventilation (P2) with desflurane will be performed after the administration of rocuronium (0.6-1 mg/kg). Once the TOF ratio reaches <2, the anesthesiologist will perform tracheal intubation (P3). The lungs of the subjects will then be ventilated with desflurane and oxygen in air (FiO2=0.5), and the tidal volume and ventilation rate will be adjusted to maintain the partial pressure of end-tidal carbon dioxide between 35 and 45 mmHg. An additional venous tube will then be secured (P4).

With the patient in the lithotomy position and under general anesthesia, the abdomen will be prepared, painted, and draped in the usual manner (P5). The uterine elevator will be inserted. A skin incision will be made (P6), and a trocar will be inserted. CO_2_ gas will be infused to create a pneumoperitoneum at a pressure of 12 mmHg (P7). After entering the abdominal cavity, the patient’s position will be changed to the Trendelenburg position. The pelvic cavity and the entire abdomen will then be examined. The bilateral pelvic sidewall triangles will be opened parallel to the infundibulopelvic ligament, and ureteral dissection will be performed. The ureteral course will be identified, and the right round and ovarian ligaments will be coagulated with a bipolar endocoagulator and cut with endoscissors. The left uterine ligament will be manipulated using the method described above. The bladder flap will be pushed, and the posterior broad ligament will be mobilized. The bilateral uterine vessels will be skeletonized, coagulated, and cut. Circumferential colpotomy will be performed by using unipolar scissors. The uterus will be removed transvaginally, and the vaginal cuff will be closed using continuous intracorporeal sutures. Hemostasis and ureteral peristalsis will be ensured, and the abdominal cavity will be irrigated with normal saline. The subcutaneous tissue and skin will be closed layer by layer.

During the procedure, the infusion rate of remifentanil and the concentration of desflurane (6.0-7.0 vol%) will be adjusted to maintain stable hemodynamic values (systolic blood pressure >80 mmHg; heart rate >45 beats/min) and an appropriate depth of anesthesia (>1 minimal alveolar concentration). If necessary, ephedrine or atropine will be administered to maintain stable hemodynamic values. All decisions will be made by a qualified anesthesiologist with at least 10 years of clinical experience.

The neuromuscular blockade will be reversed by administering sugammadex (2-5 mg/kg) at the end of the surgery, and the patient will be confirmed to be fully awake (TOF >0.9) and transferred to the postanesthetic care unit (P8).

By participating in this clinical study, the study participants will not be restricted from receiving rescue medicine required in the surgical process. If rescue medicine or rescue treatment is administered, the researcher will record the combination of therapy and rescue medicine in a case report form.

Since the intervention will be conducted over a short period, strategies for study adherence have been deemed unnecessary. However, to improve adherence, follow-up surveys will be conducted both online and offline. The online platform data are only accessible to the principal investigator and the delegated research coordinator. Other data will be electronically locked or stored in a document archive accessible only to researchers for the duration of the study and for 3 years after its completion.

### Outcome Measures

To investigate the efficacy of acupuncture on initial blood pressure fluctuations during laparoscopic hysterectomy, we will compare the difference between the highest and lowest mean blood pressures from the induction of anesthesia to the postincision period as the primary endpoint. As secondary outcomes, systolic, diastolic, and mean blood pressures will be compared at each predetermined timepoint. The incidence of hypotension (systolic blood pressure <90 mmHg or 80% of the baseline), hypertension (systolic blood pressure >160 mmHg or 120% of the baseline), tachycardia, and bradycardia will be recorded. The total use of remifentanil, surgical discontinuation, and length of hospital stay will be assessed as surrogate indicators of stable general anesthesia and surgical procedures.

For patient-reported outcomes, the Spielberger State-Trait Anxiety Inventory (STAI) and EQ-5D-5L will be used to evaluate changes in anxiety and the overall quality of life. The STAI was first validated in 1996 [16,17]. The STAI consists of 40 questions divided into 2 categories: 20 items assessing state anxiety and 20 items assessing trait anxiety. State anxiety refers to experiencing anxiety at the moment, while trait anxiety refers to the type and characteristics of anxiety.

The EQ-5D-5L will be used to assess the effects on the patient’s quality of life. The questionnaire consists of questions in 5 areas: mobility, self-care, usual activities, pain, and anxiety/depression. The EQ-5D-5L assesses the patient’s current state of health. Responses will be provided on a 5-point Likert scale [18].

**Figure 1:**
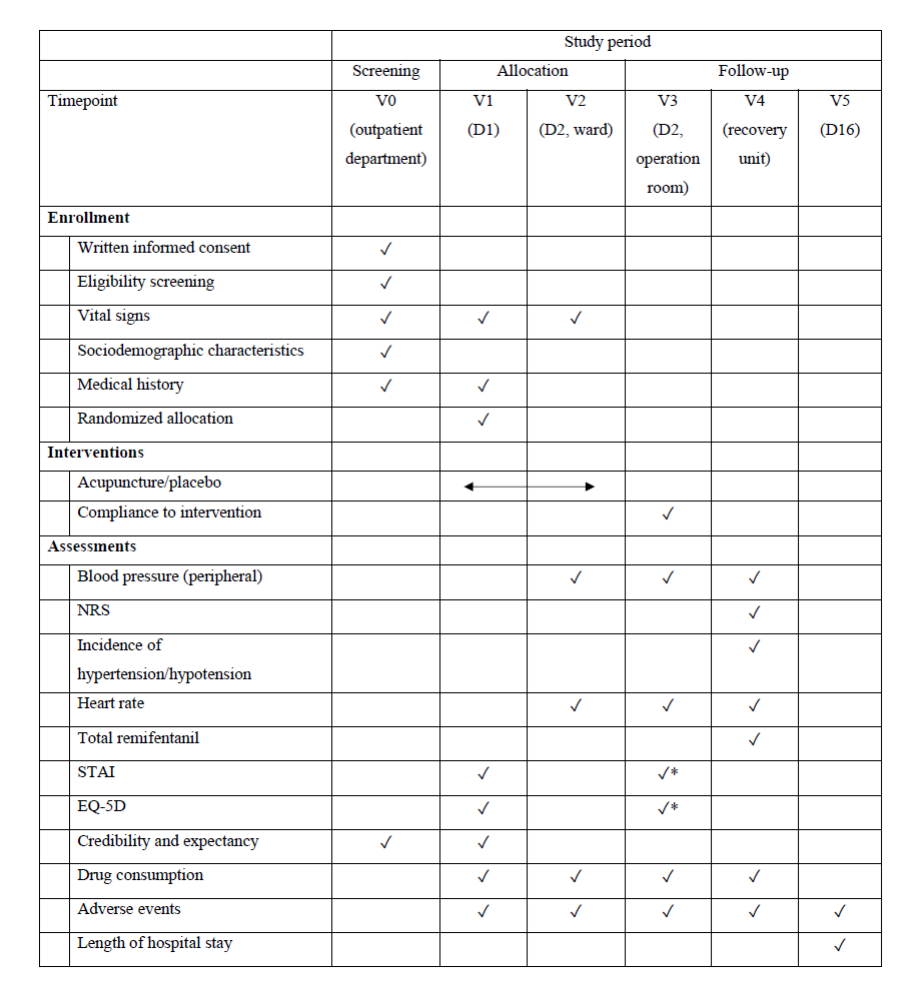
Timetable and outcome measurements. NRS: numeric rating scale; STAI: State-Trait Anxiety Inventory. *After the intervention, preoperative, and prior to anesthesia induction.

The study process and timepoints are presented in Figure 1.

### Concomitant Care

The participants are not restricted from concomitant care, similar to the conditions in an actual clinical environment. They can choose any type of combination therapy and any necessary salvage treatment throughout the hospitalization period. Researchers will document these therapies in the case report form. Documentation of these therapies will include the type, dosage, route of administration, frequency, and purpose.

### Safety

Safety will be evaluated by grading the severity of the symptoms or medical conditions. Adverse events will be defined according to MedDRA 24.0, and causality will be assessed according to the World Health Organization-Uppsala Monitoring Centre (WHO-UMC) Causality Assessment. The severity of subjective and objective symptoms will be evaluated according to the Common Terminology Criteria of Adverse Events version 5.0.

The clinical study is covered by liability insurance to compensate for any foreseeable or unforeseeable harm that may occur during the research process.

### Sample Size

No prior clinical trial has examined the effect of acupuncture on intraoperative blood pressure fluctuations. As acupuncture is applied as an adjunct to propofol-based general anesthesia in this study, sample size estimation is based on a study comparing mean arterial pressure (MAP) variability between remimazolam and propofol anesthesia [19], which reported MAP fluctuations of 26.7 (SD 9.1) mmHg and 19.5 (SD 7.5) mmHg, respectively. Although the interventions differ, this dataset was considered the best available proxy for estimating between-group differences in MAP variability under general anesthesia.

Using these values, 21 participants per group would be required to achieve 80% power with a 2-sided α of .05 when employing the *t*-test modules, considering that this study is a pilot trial. Assuming a 10% dropout rate due to the short follow-up period, the final sample size has been set at 24 participants per group, totaling 48. All calculations are performed using G*Power version 3.1.9.7 (Heinrich-Heine-University Düsseldorf).

### Statistical Analysis

Both intention-to-treat and per-protocol analyses will be performed, with intention-to-treat being the primary analysis. For per-protocol analysis, the study participants who successfully complete the surgery will be analyzed separately. Successful completion of the surgery is defined as the completion of the scheduled total laparoscopic hysterectomy under general anesthesia without protocol violations, conversion to open surgery, early termination due to intraoperative complications, or major deviations from the anesthetic protocol, such as changes in the anesthetic agents beyond the predefined regimen. For handling missing data, multiple imputations will be performed to obtain estimates and standard errors by setting 10 imputation sets.

The primary endpoint of this clinical trial is the difference between the highest and lowest mean blood pressure measured from anesthesia induction to the postincision period. The Student *t* test will be used to test whether the difference between the 2 groups in the primary endpoint is statistically significant. To account for potential confounding factors affecting the primary outcome, a multivariable linear regression analysis will be conducted. Covariates will include the placebo credibility score (at V1 and V2), anxiety levels (as measured by the STAI and EQ-5D), baseline ward blood pressure (V2), and preoperative blood pressure measured in the holding area prior to operating room entry (V3).

In addition to the primary outcome, secondary outcomes will include hemodynamic stabilization time, incidence of hypotension and hypertension, comparison of drug use, and other efficacy endpoints, including STAI scores. Differences between the 2 groups will be compared using the Student *t* test or Wilcoxon rank sum test, unless otherwise specified. The 2 groups will also be compared by estimating the ratio between the blood pressure measured at each timepoint and that measured at V2, using the blood pressure at V2 as the baseline. Because blood pressure is measured repeatedly, it is not possible to assume independence between measurements; thus, generalized estimating equations will be used. To estimate this ratio, measurements will be log-transformed and included in the model. The 2 groups will be compared using the same method for the pulse rate measured at V2 and the pulse rate at each timepoint. To quantify the length of hospitalization in both groups, we will use Kaplan-Meier estimation to estimate the time until 50% of the participants are discharged. The log-rank test will be used to determine differences in the distribution of discharge times between the 2 groups.

The significance level for all analyses is set at .05. All statistical analyses will be performed using SAS 9.4 (SAS Institute, Inc) or R software 4.1 (R Foundation for Statistical Computing).

## Results

**Figure 2:**
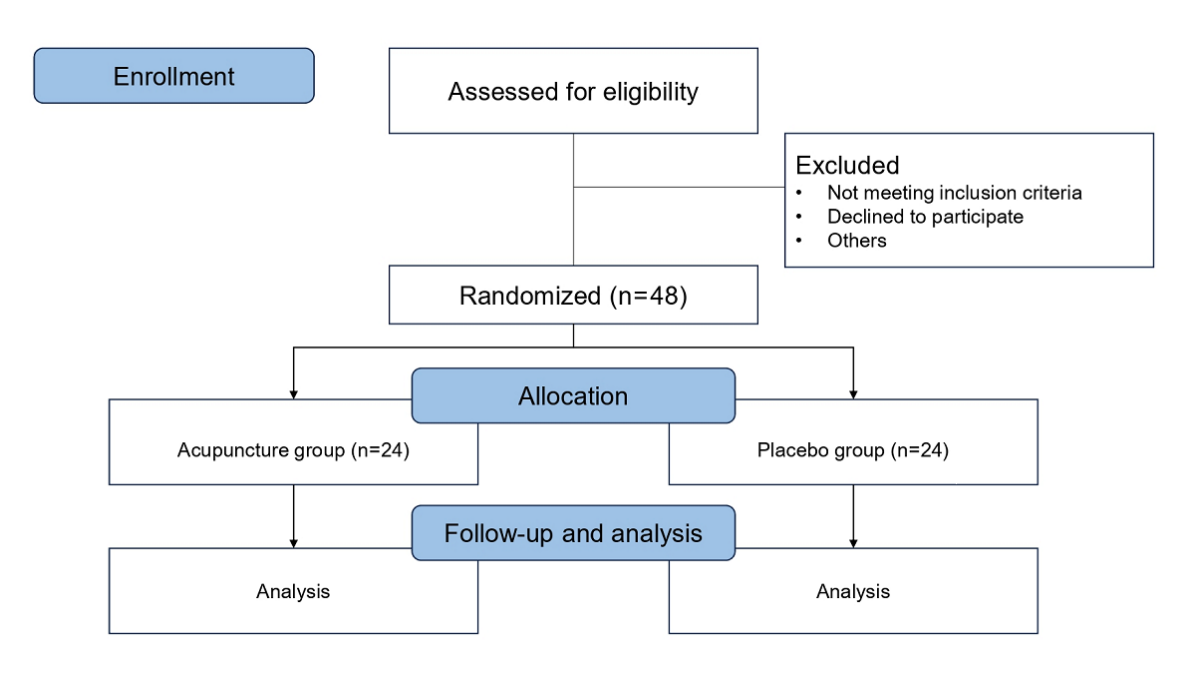
Flow diagram of the trial.

Recruitment for the study commenced in October 2023 and is ongoing, with enrollment expected to continue through December 2025. The flow diagram of the trial is shown in Figure 2. As of June 2025, a total of 35 participants have been successfully recruited and randomized. Although the intervention and data collection procedures have been proceeding without major difficulties, the overall pace of recruitment has been slower than anticipated. This is primarily due to fluctuations in surgical scheduling and staffing capacity caused by medical strikes in the Republic of Korea [20].

All participants enrolled thus far are female, reflecting the eligibility condition of requiring total laparoscopic hysterectomy. The mean age of the enrolled participants is 48.3 years. The current dropout rate is 11.4%, and up to the time of this protocol description, all reported adverse events have been mild and unrelated to the intervention.

Final data collection is projected to be completed by December 2025, which will be followed by data cleaning and statistical analysis through March 2026. Publication of the main study results is expected in the latter half of 2026.

## Discussion

The primary role of anesthesiologists during surgery extends beyond maintaining unconsciousness to actively minimizing the physical stress induced by intraoperative stimuli. These stimuli include not only surgical manipulation but also the administration of anesthetics and analgesics, which, while essential, can disrupt homeostasis and result in intraoperative hypotension. Factors, such as surgical bleeding and patient positioning, may exacerbate this effect. Even in patients without comorbidities, sustained hypotension has been associated with poor postoperative outcomes [21,22], and hypertensive episodes during surgery, often a sign of inadequate analgesia, are similarly linked to increased risks of major adverse cardiovascular events [3]. Thus, maintaining blood pressure within a target range is an important goal of anesthetic management.

Laparoscopic hysterectomy is a procedure often associated with significant hemodynamic fluctuations, particularly in the early intraoperative period. Pneumoperitoneum and Trendelenburg positioning may stimulate sympathetic activation or trigger vasovagal reflexes, resulting in rapid changes in blood pressure [15,23,24]. Anesthesiologists must anticipate nociceptive stimuli, such as tracheal intubation and skin incision, and preemptively adjust opioid administration to ensure hemodynamic stability [25,26]. However, due to interindividual variability and the pharmacokinetics of agents like remifentanil, achieving consistent control is challenging [27,28]. Although remifentanil’s rapid onset and offset allow precise titration, excessive dosing has been linked to adverse effects such as delayed emergence, postoperative nausea and vomiting, and opioid-induced hyperalgesia [29]. Because of these dose-related risks and the challenge of achieving consistent hemodynamic control, minimizing remifentanil use without compromising analgesia is a meaningful anesthetic goal. Reflecting this, the amount of remifentanil administered during the early surgical phase has been included as a secondary outcome in our study, aligning with the broader goal of minimizing opioid use while preserving analgesic efficacy [30,31]

Acupuncture has been proposed as a supportive technique to anesthesia, with studies reporting its potential to reduce anesthetic requirements, alleviate perioperative anxiety, and improve postoperative recovery [32,33]. Given its low invasiveness and favorable safety profile (characterized by mostly minor local reactions and rare treatment-requiring events), acupuncture remains a feasible adjunct in the perioperative setting [34]. Although its role in intraoperative blood pressure regulation remains under investigation, emerging evidence points to its influence on autonomic nervous system modulation. Functional magnetic resonance imaging studies have demonstrated that acupuncture alters hypothalamic connectivity, a key brain region involved in cardiovascular control [13]. Additionally, acupuncture has been shown to attenuate hemorrhage-induced hypotension in animal models and lower systemic blood pressure in patients with hypertension [10,35,36].

Outside the perioperative setting, acupuncture has shown clinically meaningful effects in reducing blood pressure among patients with essential hypertension. For example, studies by Flachskampf et al [35] and Yin et al [36] reported significant reductions in systolic and diastolic blood pressure after 6 to 8 weeks of repeated acupuncture sessions [10], whereas studies with shorter durations (15-25 days) failed to demonstrate such benefits [37]. Overall, although the long-term use of acupuncture therapy has blood pressure–lowering effects of 4-10 mmHg in patients with uncontrolled hypertension, the effects of short-term acupuncture therapy on blood pressure fluctuations have not yet been clearly established.

One important limitation in previous studies assessing acupuncture as an anesthetic adjunct is the timing and duration of stimulation [38]. Acupuncture requires adequate time to induce physiological effects, with analgesic effects typically emerging 20 minutes after needle insertion and lasting for just several hours. Therefore, acupuncture initiation only shortly before or during anesthesia may have different physiological potential, particularly because volatile anesthetics can suppress neural pathways responsive to acupuncture.

In this context, the selection of acupoints in the treatment group was based on previous studies demonstrating their physiological relevance in autonomic modulation and cardiovascular regulation. Specifically, PC5, PC6, and KI3 have been widely associated with reductions in perioperative anxiety and stabilization of heart rate variability, while ST36 is well-documented for its influence on vagal activation and blood pressure regulation. SP3 has been included for its role in supporting systemic homeostasis. For the selection of placebo acupoints, the following 3-fold strategy has been considered to minimize specific physiological stimulation: (1) if possible, a sham needle is preferable; (2) points should avoid all recognized World Health Organization acupoints and should not be situated along any known meridian pathways; and (3) anatomical regions are restricted to outside the limbs, where the treatment acupoints are applied. Since the use of nonpenetrating sham needles is not possible due to product discontinuation, the other 2 strategies have been used for the placebo group.

Our study protocol was designed to optimize the timing of acupuncture application, administering stimulation at 2 timepoints, once on the day before surgery and again on the morning of the surgery, which would allow a sufficient lead time before anesthesia induction. This schedule was chosen based on both clinical feasibility and physiological rationale. As patients are routinely admitted the day before laparoscopic hysterectomy, it is practical to deliver acupuncture during the hospital stay without requiring additional outpatient visits. Moreover, this repeated, preoperative approach provides a longer stimulation window than the approach in most prior studies evaluating acupuncture as an anesthetic adjunct, which typically applied stimulation only shortly before or during anesthesia. While the duration remains shorter than long-term outpatient regimens used for chronic hypertension, it represents a balanced and realistic timeframe to allow acupuncture’s physiological effects to manifest before the influence of general anesthetics begins, potentially enhancing its effectiveness in modulating intraoperative hemodynamics.

Another strength of our design is the inclusion of psychological covariates to explore individual variability in response. Although not a primary or secondary endpoint, researchers have incorporated validated tools, such as the Spielberger STAI and EQ-5D-5L, to assess baseline anxiety and quality of life. These tools enable exploratory analysis of whether the psychological state moderates the physiological effects of acupuncture, helping to distinguish autonomically mediated responses from those influenced by psychological factors. This addresses a common limitation of prior acupuncture trials, which often did not account for patient-level psychological differences.

For our primary outcome, researchers chose absolute thresholds of MAP, rather than relative changes, due to inconsistencies in baseline blood pressure definitions in perioperative settings. Establishing a reliable baseline for relative changes is challenging, as preinduction blood pressure often differs from values obtained during preoperative evaluation [38], and normal blood pressure levels can vary depending on individual conditions and circadian rhythms [39]. Given these limitations, researchers have adopted standardized MAP thresholds: values below 65 mmHg are associated with impaired organ perfusion, while sustained elevations above 80 mmHg may increase the risk of major adverse cardiovascular events [40]. These thresholds align with intraoperative blood pressure management goals, providing a clear rationale for selecting them as our primary outcome measure. This standardization, along with the use of 2.5-minute noninvasive blood pressure monitoring, enables close tracking of blood pressure changes during the highly dynamic early intraoperative phase [41].

Despite its strengths, our study protocol has several limitations. First, as with most acupuncture trials, blinding of the practitioner is not feasible. In this study, however, acupuncture is administered preoperatively by a certified traditional medicine physician in the ward, while intraoperative management and outcome assessments are independently performed by anesthesiologists in the operating room. This structural separation minimizes the risk of performance and detection bias. Additionally, outcome assessors remain blinded to group allocation throughout the trial. Second, this is a single-center exploratory study, which may limit the generalizability of the findings. Nevertheless, the trial’s methodological strengths, including the timing and repetition of acupuncture, the incorporation of psychological covariates, and the focused monitoring of intraoperative blood pressure, enhance its internal validity and potential clinical relevance.

In summary, this randomized controlled trial is designed to evaluate whether preoperative acupuncture, applied at 2 timepoints, can help stabilize intraoperative blood pressure during laparoscopic hysterectomy. By addressing both physiological mechanisms and psychological factors, the study aims to clarify the short-term effects of acupuncture on hemodynamic stability in a controlled surgical setting. The results may provide useful evidence on the feasibility and potential benefits of incorporating acupuncture into perioperative care to reduce blood pressure fluctuations and minimize opioid requirements.

## Appendix

Multimedia Appendix 1 SPIRIT (Standard Protocol Items: Recommendations for Interventional Trials) checklist.

Multimedia Appendix 2 STRICTA (Standards for Reporting Interventions in Clinical Trials of Acupuncture) checklist.

## Abbreviations

DSMB: data and safety monitoring board
MAP: mean arterial pressure
STAI: State-Trait Anxiety Inventory
TOF: train of four

## Data Availability

Publication is scheduled for December 2026. The data will not be publicly available but can be requested from the corresponding author.
