# Correction: Rationale for the Use of Acupuncture to Stabilize Blood Pressure Fluctuations During Total Laparoscopic Hysterectomy: Protocol for a Pilot Parallel-Group Randomized Clinical Trial

**DOI:** 10.2196/82570

**Published:** 2025-09-23

**Authors:** Jee Young Lee, Ju-Won Roh, Kyung-Hee Han, Min-Jeong Kim, Young Jeong Na, Bo Seong Yun, Joohyun Lee

**Affiliations:** 1 Department of Korean Medicine, Integrative Cancer Center, CHA Ilsan Medical Center, CHA University Goyang-si Republic of Korea; 2 Department of Obstetrics and Gynecology, CHA Ilsan Medical Center, CHA University Goyang-si Republic of Korea; 3 Seoul National University Hospital Seoul Republic of Korea; 4 Department of Anesthesia and Pain Medicine CHA Ilsan Medical Center CHA University Goyang-si Republic of Korea

In “Rationale for the Use of Acupuncture to Stabilize Blood Pressure Fluctuations During Total Laparoscopic Hysterectomy: Protocol for a Pilot Parallel-Group Randomized Clinical Trial” ([JMIR Res Protoc 2025;14:e77009]) the authors noted one error.

Under the Acknowledgments section, the phrase:

...(grant number: RS-2023-KH139140 [HF23C0097]).

has been replaced by:

...(grant number: RS-2023-KH139468 [HF23C0097]).

The updated Acknowledgments section now appears as follows:

This research is supported by a grant from the Korea Health Technology R&D Project through the Korea Health Industry Development Institute, funded by the Ministry of Health & Welfare, Republic of Korea (grant number: RS-2023-KH139468 [HF23C0097]). The funders had no role in the study design, data collection, analysis, interpretation, manuscript writing, or decision to publish the results.

The correction will appear in the online version of the paper on the JMIR Publications website, together with the publication of this correction notice. Because this was made after submission to PubMed, PubMed Central, and other full-text repositories, the corrected article has also been resubmitted to those repositories.

