# Supplementary material for: Rationale for the Use of Acupuncture to Stabilize Blood Pressure Fluctuations During Total Laparoscopic Hysterectomy: Protocol for a Pilot Parallel-Group Randomized Clinical Trial
Source: JMIR Res Protoc. 2025 Jul 22;14:e77009. doi: 10.2196/77009 (PMC12326159; doi:10.2196/77009)
Supplement: Multimedia Appendix 2 [file resprot_v14i1e77009_app2.pdf]

Multimedia Appendix 2. STRICTA (Standards for Reporting Interventions in Clinical Trials of Acupuncture) checklist

STRICTA 2010 checklist of information to include when reporting interventions in a clinical trial of acupuncture (Expansion of Item 5 from CONSORT 2010 checklist)

Item	Detail	Page
1. Acupuncture Rationale	1a) Style of acupuncture (e.g. Traditional Chinese Medicine, Japanese, Korean, Western medical, Five Element, ear acupuncture, etc)	13
	1b) Reasoning for treatment provided, based on historical context, literature sources, and/or consensus methods, with references where appropriate	13
	1c) Extent to which treatment was varied	7
2. Details of needling	2a) Number of needle insertions per subject per session (mean and range where relevant)	7
	2b) Names (or location if no standard name) of points used (uni/bilateral)	7
	2c) Depth of insertion, based on a specified unit of measurement, or on a particular tissue level	7
	2d) Response sought (e.g. de qi or muscle twitch response)	7
	2e) Needle stimulation (e.g. manual, electrical)	7
	2f) Needle retention time	7
	2g) Needle type (diameter, length, and manufacturer or material)	7
3. Treatment regimen	3a) Number of treatment sessions	7
	3b) Frequency and duration of treatment sessions	7
4. Other components of treatment	4a) Details of other interventions administered to the acupuncture group (e.g. moxibustion, cupping, herbs, exercises, lifestyle advice)	7
	4b) Setting and context of treatment, including instructions to practitioners, and information and explanations to patients	7
5. Practitioner background	5) Description of participating acupuncturists (qualification or professional affiliation, years in acupuncture practice, other relevant experience)	7
6. Control or comparator interventions	6a) Rationale for the control or comparator in the context of the research question, with sources that justify this choice	7
	6b) Precise description of the control or comparator. If sham acupuncture or any other type of acupuncture-like control is used, provide details as for Items 1 to 3 above.	7

Citation: MacPherson H, Altman DG, Hammerschlag R, Youping L, Taixiang W, White A, Moher D; STRICTA Revision Group. Revised STandards for Reporting Interventions in Clinical Trials of Acupuncture (STRICTA): extending the CONSORT statement. PLoS Med. 2010 Jun 8;7(6):e1000261
